# Pectoralis Major Rupture in a 15-year-old Teenager: Case Report

**DOI:** 10.1055/s-0044-1785448

**Published:** 2024-05-19

**Authors:** Paulo César Faiad Piluski, João Artur Bonadiman, Eduardo Necher Moreira, Carlos Humberto Castillo Rodriguez, Osvandré Lech

**Affiliations:** 1 Instituto de Ortopedia e Traumatologia/Hospital São Vicente de Paulo, Passo Fundo, RS, Brasil; 2 Instituto Brasil de Tecnologias da Saúde, Rio de Janeiro, RJ, Brasil

**Keywords:** pectoralis muscles, shoulder/surgery, sports medicine, tendon injuries

## Abstract

Rupture of the pectoralis major muscle is extremely rare in adolescents. The current literature contains only 5 reports of this condition in patients under 20 years old, with 2 reports in subjects under 16. In the present article, we report the case of a 15-year-old volleyball player who suffered a traumatic rupture of the pectoralis major in a match during the serve movement. After excluding endocrinological abnormalities as a cause of tendon weakening, the patient underwent surgical treatment due to muscle retraction, strength deficit, high demand, and esthetic concerns. Early diagnosis is crucial to successful outcomes, and the surgical intervention enabled early rehabilitation and a provided a higher likelihood of return to high-level competitive sports.

## Introduction

Rupture of the pectoralis major muscle is an uncommon injury that occurs| more frequently in adults between 20 and 40 years old. 1 The main causes of pectoralis major muscle rupture include weight lifting (particularly bench press), followed by trauma resulting from football, water skiing, wrestling, and ice hockey. 2 Although this injury predominantly affects male individuals, women have been gradually increasing their participation in these predisposing sports. 3 4 Furthermore, this condition is considered extremely rare in adolescents, an age group with higher elasticity, in whom the muscles are still developing, and muscle strength is often lower than in adults. 5 As far as we know, the literature has only two reports 6 7 of pectoralis rupture in patients aged 16 or younger.

Despite its clinical relevance, virtually all studies on this injury focus on adults, which further highlights the rarity of this condition in female adolescents and the need for more research in this field. The objective of the present article is to review the literature currently available on rupture of the pectoralis major in adolescents, as well as to report a case of a 15-year-old female patient, a volleyball player at a high-demand school competitive level who suffered a traumatic rupture of the pectoralis major during a volleyball game and underwent surgical treatment.

## Case Report

The institutional Review Board approved the present case report. The patient's legal guardian signed the informed consent form.

A 15-year-old female patient with no history of illness presented with a clinical picture of abrupt pain in the right pectoral region after executing a serve movement during a volleyball game 7 days ago. The physical examination revealed ecchymosis in the area corresponding to the right pectoral region and a palpable gap close to the axillary midline. The passive range of motion was preserved, with painful complaints at the end of the adduction and internal rotation movements of the right upper limb. The strength of adduction and internal rotation of the right upper limb was of grade 3, with sustained strength in other movements. The patient presented no neurological deficits. According to the Risser scale, the degree of skeletal maturation was stage 5. Regarding the development of secondary sexual characteristics, the patient was in Turner stages M3 and P4, with menarche occurring 1 year before.

**Fig. 1:**
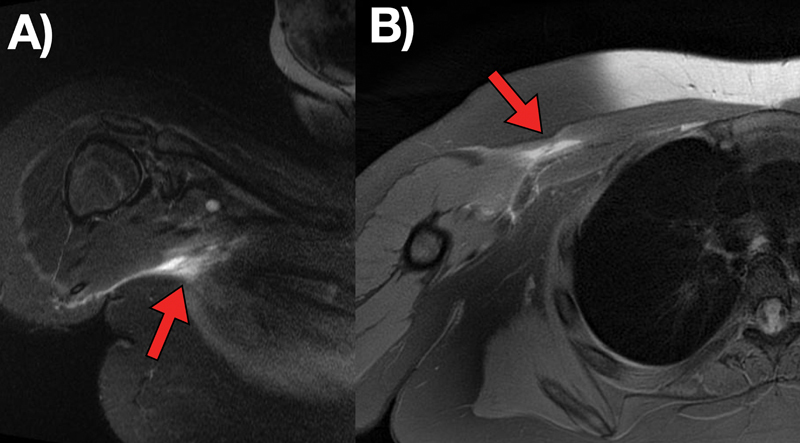
T2-weighted magnetic resonance imaging scans showing rupture of the pectoralis major muscle in the axial ( **A** ) and oblique coronal ( **B** ) sections, with details of retraction (red arrow).

Standard radiographs showed no changes. Next, magnetic resonance imaging (MRI) revealed extensive rupture in the distal myotendinous transition in the pectoralis major, mainly affecting the sternal portion, with signs of distal stump retraction ( Fig. 1 ).

**Fig. 2:**
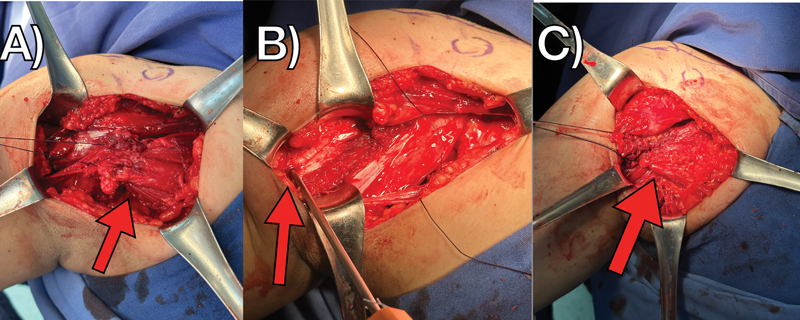
Intraoperative images showing injury to the pectoralis major tendon with retraction ( **A** ), insertion of a metal anchor next to the footprint of the pectoralis major ( **B** ), and the final appearance after reattachment ( **C** ).

**Fig. 3:**
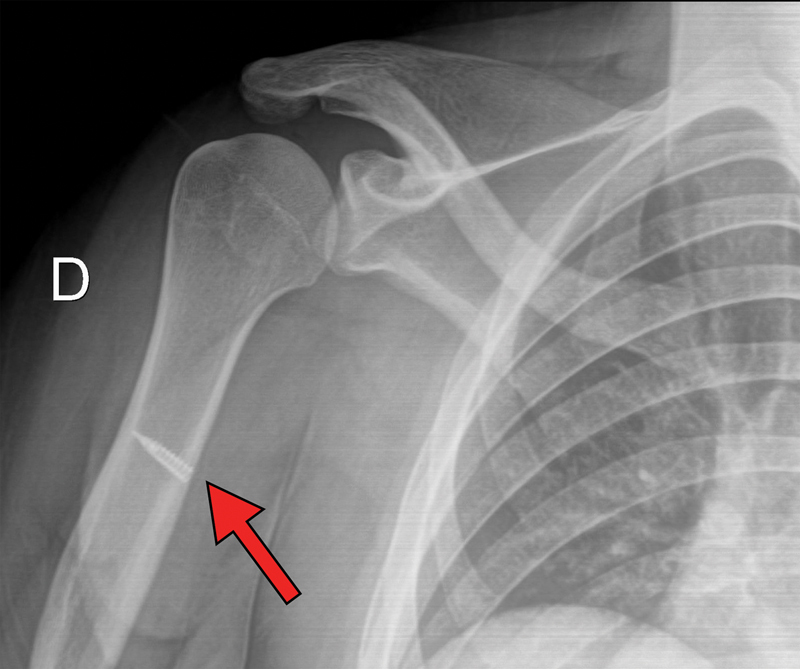
Follow-up radiograph of the position of the metallic anchor (red arrow).

**Fig. 4:**
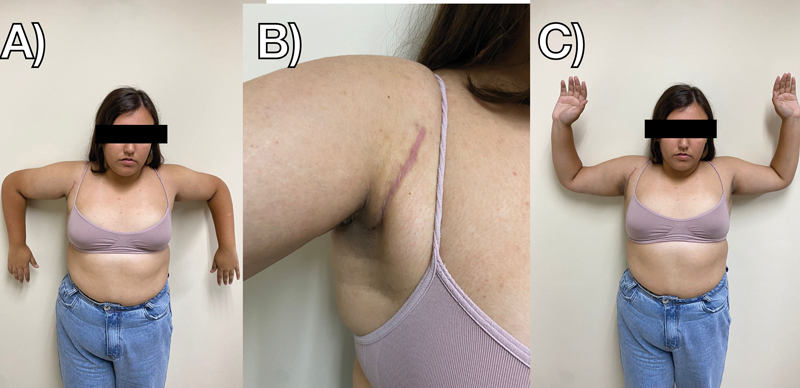
Postoperative clinical follow-up after 15 weeks. ( **A** ) Internal rotation. ( **B** ) Surgical scar. ( **C** ) External rotation.

After explaining the therapeutic options to the patient and her parents, surgical treatment was chosen and performed eight days after the first visit. During the operation, pectoralis major rupture was evident, with retraction of the sternal belly, and only a few fibers remaining in the native attachment ( Fig. 2A ). After adequate exposure, we inserted a 5.5-mm Twinfix (Smith & Nephew, Watford, Hertfordshire, United Kingdom) titanium metal anchor double loaded with Ultrabraid wires (Smith & Nephew) into the footprint of the pectoralis major tendon, next to the medial surface of the humerus ( Fig. 2B ), and the repair was performed with Krakow sutures on the distal stump of the pectoralis major, which brought the tendon close to the footprint, restoring physiological tension and length ( Fig. 2C ). After two weeks of immobilization with a sling, a follow-up radiograph revealed the position of the anchor ( Fig. 3 ), and the patient started pendulum exercises and physical therapy for passive mobility. Six weeks after surgery, the patient stopped using the sling, and physical therapy progressed to active mobility exercises. Gradual muscle-strengthening exercises started after ten weeks. At that moment, laboratory tests investigated the levels of thyroid-stimulating hormone (THS), free thyroxine (T4), triiodothyronine (T3), follicle-stimulating hormone (FSH), luteinizing hormone (LH), progesterone, prolactin, estradiol, and antidiuretic hormone (ADH), which were within normal limits. We requested these tests to exclude any endocrinological abnormalities that could justify the occurrence of this uncommon lesion regarding the patient's profile and age range, since certain endocrinological conditions, such as hyperthyroidism, hypogonadism, and hyperparathyroidism, may result in myotendinous lesions. 8 After 15 weeks, the patient returned for reevaluation; she was asymptomatic, presenting good muscle contour, complete range of motion, grade-4 strength for adduction and internal rotation, and grade-5 for other movements (clinically assessed by the examiner), and an American Shoulder and Elbow Surgeons (ASES) score of 90 (it was of 28 before surgery) ( Fig. 4 ). We advised the patient to progress with gym activities and return to volleyball after completing 20 weeks of surgery. Six months after the procedure, the patient returned for a final follow-up visit, asymptomatic, with physiological muscle contour, and a complete return to volleyball activities, with no limitations and an ASES score of 94.

## Discussion

Rupture of the muscle of the pectoralis major tendon is extremely rare in this patient's age group. Pochini et al. 9 studied 27 patients with pectoralis major rupture; the average age was 29.9 years old, with the youngest subject being 21 years old. 9 In another study by the same group of authors, 10 with a sample of 60 cases, the youngest patient was also 21 years old. In the systematic review of 365 cases by ElMaraghy and Deveraux, 11 the average age was of approximately 31 years, with a standard deviation of 9.9 years. Moreover, this study 11 revealed that, out of the 365 cases analyzed, only 11 occurred in women. The systematic review by Yu et al. 12 included 536 cases of pectoralis major rupture; the mean age was of 28 ± 3 years, and there was only one female subject. These data highlight the rarity of this condition in adolescent and female patients. There are only 5 reports 6 7 13 14 15 in the literature with patients under 20 with this type of injury. Virtually in all cases, the patients practiced some sport, such as tennis, 6 wrestling, 7 judo, 13 and softball. 15 In the case herein reported, the 15-year-old teenager played volleyball at a school competition level. It is worth mentioning that, according to Reeser et al., 16 63% of volleyball players present some degree of shortening of the pectoral muscles. This factor may have contributed to the injury in question, since laboratory tests ruled out endocrinological changes.

The option for surgical treatment was based on the high-demand activity of the patient, the retraction associated with strength deficit, and cosmetic concerns. Several studies advocate that surgical intervention improves strength and cosmesis, in addition to enabling the athlete to return to preinjury levels. 2 9 10 13 Early detection of this injury is essential to obtain good outcomes and high satisfaction levels. 10 17

The present article aimed to report a rare case of rupture of the pectoralis major muscle in an adolescent (an unusual age group for this condition), an injury whose incidence has increased in recent years. Due to the rarity of this condition, it is crucial to always suspect it for an accurate and timely diagnosis. Early diagnosis can enable rapid surgical intervention aiming at early rehabilitation and greater predictability of return to sport, as demonstrated in our patient. We hope future investigations will address appropriate treatments for adolescent athletes along with conditions and mechanisms to prevent this injury.
