# Supplementary material for: Pectoralis Major Rupture in a 15-year-old Teenager: Case Report
Source: Rev Bras Ortop (Sao Paulo). 2024 May 19;59(Suppl 2):e199–202. doi: 10.1055/s-0044-1785448 (PMC11679620; doi:10.1055/s-0044-1785448)
Supplement: Related texts

# Research article: Ruptura do peitoral maior em adolescente de 15 anos: Relato de caso

**Keywords:** lesões dos tendões, medicina esportiva, músculos peitorais, ombro/cirurgia

## Abstract

A ruptura do músculo peitoral maior é extremamente rara em adolescentes. Na literatura atual, existem apenas 5 relatos em pacientes com menos de 20 anos, sendo 2 em pacientes com menos de 16 anos de idade. Neste artigo, relatamos o caso de uma jogadora de vôlei de 15 anos que sofreu uma ruptura traumática do peitoral maior em uma partida durante o movimento de saque. Após a exclusão de causas endocrinológicas – que poderiam ter provocado o enfraquecimento tendíneo –, o tratamento cirúrgico foi escolhido devido à retração muscular, ao déficit de força, à alta demanda e às preocupações estéticas. O diagnóstico precoce é crucial para que se obtenham bons resultados, e a intervenção cirúrgica propiciou a reabilitação precoce e uma maior probabilidade de retorno ao esporte de competição em alto nível.

## Introdução

A ruptura do músculo peitoral maior é uma lesão incomum que ocorre mais frequentemente em adultos na faixa etária entre 20 e 40 anos. 1 Entre as principais causas desse tipo de lesão, o levantamento de peso (particularmente o supino) é o mecanismo mais comum, seguido de traumas decorrentes de futebol, esqui aquático, luta livre e hóquei no gelo. 2 Essa lesão acomete predominantemente indivíduos do sexo masculino, embora a adesão a esses esportes predisponentes venha aumentando gradativamente entre indivíduos do sexo feminino. 3 4 Além disso, essa condição é considerada extremamente rara em adolescentes, uma faixa etária com maior elasticidade, em que a musculatura ainda está em desenvolvimento, e cuja força muscular geralmente é menor em comparação com adultos. 5 Pelo que sabemos, existem apenas 2 relatos na literatura 6 7 sobre ruptura do peitoral maior em pacientes com 16 anos de idade ou menos.

Apesar de sua relevância clínica, quase a totalidade dos estudos sobre essa lesão se concentra em adultos, o que ressalta ainda mais a raridade dessa condição em adolescentes do sexo feminino e a necessidade de mais pesquisas nessa área. O objetivo deste artigo é revisar a literatura atualmente disponível sobre a ruptura do peitoral maior em adolescentes, bem como relatar um caso de paciente do sexo feminino, de 15 anos, praticante de vôlei em nível competitivo escolar de alta demanda, com ruptura traumática do peitoral maior durante uma partida, submetida ao tratamento cirúrgico.

## Relato de Caso

Este relato de caso foi aprovado pelo Comitê de Revisão institucional, e o responsável pela paciente assinou o termo de consentimento livre e esclarecido.

Uma paciente do sexo feminino, de 15 anos de idade e sem histórico de doenças, apresentou quadro clínico de dor abrupta na região peitoral direita após executar movimento de saque durante uma partida de vôlei havia 7 dias. Durante o exame físico, foi observada a presença de equimose na área correspondente ao peitoral direito, assim como a presença de *gap* palpável próximo à linha média axilar. A amplitude movimento passiva estava preservada, e a paciente apresentava queixa dolorosa ao final da mobilidade de adução e rotação interna do membro superior direito. A força de adução e rotação interna do membro superior direito foi classificada como de grau 3, ao passo que a força nos demais movimentos estava preservada. A paciente não apresentava déficits neurológicos. De acordo com a escala de Risser, a maturação esquelética era classificada como de estágio 5. Quanto ao desenvolvimento dos caracteres sexuais secundários, foram observados os estágios M3 e P4 de Turner, com a menarca ocorrida havia 1 ano.

Foram realizadas radiografias padrão, que não apresentaram alterações. Em seguida, realizou-se ressonância magnética, que evidenciou extensa rotura na transição miotendínea distal no peitoral maior, que acometia principalmente a porção esternal, com sinais de retração do coto distal ( Fig. 1 ).

**Fig. 1:** Imagens de ressonância magnética ponderadas em T2 que mostram ruptura do músculo peitoral maior nos cortes axial ( **A** ) e coronal oblíquo ( **B** ), com detalhes da retração (seta vermelha).

Após explanar as opções terapêuticas para a paciente e os seus pais, optou-se pelo tratamento cirúrgico, o qual foi realizado oito dias após a primeira consulta. No transoperatório, evidenciou-se ruptura do peitoral maior com retração do ventre esternal, com apenas algumas fibras remanescentes na inserção nativa. ( Fig. 2A ). Após a realização da exposição adequada, foi inserida uma âncora metálica de titânio de 5.5 mm Twinfix (Smith & Nephew, Watford, Hertfordshire, Reino Unido) duplamente carregada com fios Ultrabraid (Smith & Nephew,) no *footprint* do tendão peitoral maior, junto à face medial do úmero ( Fig. 2B ), e fez-se o reparo com pontos do tipo Krakow no coto distal do peitoral maior, o que aproximou o tendão do *footprint* , assim restaurando a tensão e o comprimento fisiológicos ( Fig. 2C ). Após duas semanas de imobilização com tipoia, solicitamos radiografia de controle para verificar o posicionamento da âncora ( Fig. 3 ), e a paciente iniciou exercícios pendulares e fisioterapia para a mobilidade passiva. Seis semanas após a cirurgia, o uso da tipoia foi descontinuado, e a fisioterapia progrediu para exercícios de mobilidade ativa. Exercícios de reforço muscular gradativo foram iniciados após 10 semanas, quando também foi realizada investigação laboratorial com dosagem de hormônio estimulante da tireoide ( *thyroid-stimulating hormone* , TSH, em inglês), tiroxina (T4) livre, tri-iodotironina (T3), hormônio folículo-estimulante ( *follicle-stimulating hormone* , FSH, em inglês), hormônio luteinizante ( *luteinizing hormone* , LH, em inglês), progesterona, prolactina, estradiol e hormônio antidiurético ( *antidiuretic hormone* , ADH, em inglês), os quais apresentaram-se dentro dos limites da normalidade. Solicitamos esses exames visando excluir alguma alteração endocrinológica que justificasse essa lesão fora do perfil e da faixa etária, visto que algumas condições endocrinológicas, como hipertireoidismo, hipogonadismo e hiperparatireoidismo, podem estar relacionadas ao surgimento de lesões miotendíneas. 8 Após 15 semanas, a paciente retornou para reavaliação, assintomática, com bom contorno muscular, amplitude de movimento completa, força de grau 4 para adução e rotação interna, e de grau 5 para os demais movimentos (aferidos clinicamente pelo examinador) com pontuação de 90 na classificação da American Shoulder and Elbow Surgeons (ASES; era de 28 antes da cirurgia) ( Fig. 4 ). A paciente foi orientada a progredir com atividades na academia e a retornar ao vôlei após completar 20 semanas da cirurgia. Com seis meses do pós-operatório, a paciente retornou para a reavaliação final, assintomática, com contorno muscular fisiológico e retorno completo às atividades de vôlei sem limitações, e pontuação da ASES de 94.

**Fig. 2:** Imagens do transoperatório que mostram lesão do tendão peitoral maior com retração ( **A** ), inserção de âncora metálica junto ao *footprint* do peitoral maior ( **B** ) e aspecto final após reinserção ( **C** ).

**Fig. 3:** Radiografia de controle do posicionamento da âncora metálica (seta vermelha).

**Fig. 4:** Controle clínico pós-operatório após 15 semanas. ( **A** ) Rotação interna. ( **B** ) Cicatriz operatória. ( **C** ) Rotação externa.

## Discussão

A ruptura do músculo do tendão do peitoral maior é extremamente rara na faixa etária da paciente deste relato de caso. No estudo de Pochini et al., 9 o qual contou com uma amostra de 27 pacientes com ruptura do peitoral maior, a faixa etária média foi de 29,9 anos, e o paciente mais jovem tinha 21 anos. 9 Em outro estudo do mesmo grupo de autores 10 (grupo de 60 casos), o paciente mais jovem também tinha 21 anos. Na revisão sistemática conduzida por ElMaraghy e Deveraux, 11 em que foram analisados 365 casos, constatou-se que a média de idade girava em torno de 31 anos, com um desvio padrão de 9.9 anos. Além disso, esse estudo 11 revelou que, dos 365 casos analisados, apenas 11 ocorreram em mulheres. A revisão sistemática de Yu et al. 12 incluiu 536 casos de ruptura do peitoral maior, na faixa etária média de 28 ± 3 anos, e apenas 1 caso do sexo feminino. Esses dados destacam a raridade dessa condição em pacientes adolescentes e do sexo feminino. Existem apenas 5 relatos na literatura 6 7 13 14 15 de pacientes com menos de 20 anos de idade com esse tipo de lesão. Em praticamente todos os casos, os pacientes já estavam envolvidos de alguma maneira com a prática de esportes, como tênis, 6 luta livre, 7 judô 13 e *softball* . 15 Neste caso em particular, a adolescente de 15 anos é praticante de vôlei em nível de competição escolar. Vale ressaltar que, conforme o estudo de Reeser et al., 16 63% dos jogadores de vôlei apresentam algum grau de encurtamento da musculatura peitoral, fator que pode ter contribuído para a lesão em questão, visto que alterações endocrinológicas foram descartadas pelos exames laboratoriais.

A opção pelo tratamento cirúrgico baseou-se no fato de a paciente apresentar alta demanda, retração associada a déficit de força, além da preocupação com a cosmese. Diversos estudos defendem que a intervenção cirúrgica melhora a força, a cosmese e, principalmente, possibilita que o atleta retorne aos níveis pré-lesão. 2 9 10 13 A detecção precoce dessa lesão é fundamental para a obtenção de bons resultados e altos graus de satisfação. 10 17

Este artigo teve como objetivo relatar um caso raro de ruptura do músculo peitoral maior em uma adolescente (faixa etária incomum para esse tipo de afecção), uma lesão cuja incidência vem aumentando nos últimos anos. Devido à raridade dessa condição, é crucial sempre tê-la como suspeita para o diagnóstico preciso e oportuno. O diagnóstico precoce pode permitir uma intervenção cirúrgica rápida com o objetivo de reabilitação precoce e maior previsibilidade de retorno ao esporte, o que foi demonstrado em nossa paciente. Espera-se que investigações futuras possam abordar tratamentos apropriados para adolescentes atletas, bem como condições e mecanismos de prevenção dessa lesão.
